# Informal Caregivers in Transitional Hospital-to-Home Care for Older People: Addressing Gaps in Pre-Discharge Collaboration for Enhanced Patient Outcomes

**DOI:** 10.2147/PPA.S532419

**Published:** 2025-10-11

**Authors:** Filipa Pereira, Catherine Bolduc, Pauline Melly, Virginie Renggli

**Affiliations:** 1 School of Health Sciences, HES-SO Valais-Wallis, Sion, Switzerland; 2 Valais Romand Hospital Centre, Sion, Switzerland

**Keywords:** informal caregivers, hospital-to-home transition, discharge planning, patient-centered care, caregiver collaboration

## Abstract

With aging populations worldwide, informal caregivers (ICs) play an essential role in transitional hospital-to-home care for older people, providing essential first-line support, clinical assistance, emotional care, and crucial coordination of care. However, their involvement in discharge planning remains insufficiently recognized, potentially compromising patient safety and continuity of care. Despite their vital contributions and growing policy initiatives, ICs’ involvement in hospital discharge planning remains insufficiently recognized. This lack of structured integration leads to significant gaps in communication, education, and collaborative care planning—factors that can compromise patient safety, disrupt continuity of care, and increase caregiver burden. ICs often report feeling unheard and excluded from key decisions, which contributes to adverse outcomes for both patients and ICs. Addressing these gaps requires comprehensive systemic changes aimed at formalizing the role of ICs in professional training, hospital discharge protocols, and integrating ICs meaningfully into care decision-making processes. This perspectives article provides an overview of the current state of IC involvement in transitional care, reflects on its relevance among healthcare professionals, policymakers, and researchers, and explores the implications for patient safety and health outcomes. Drawing on evidence-based models such as the Transitional Care Model and Better Outcomes for Older people through Safe Transitions, the article discusses key strategies to enhance IC participation. These include raising awareness among professional caregivers, improving communication and information transfer, formally recognizing and integrating ICs as care partners, and leveraging technology and support systems tailored to their needs. By fostering structured partnerships and collaborative approaches with ICs, healthcare systems can improve the quality and safety of transitional care while alleviating caregiver burden and promoting better long-term health trajectories for older people.

## Informal Caregivers are Essential Partners in Healthcare Systems

As worldwide demographics shift towards increasingly aged populations, the role of informal caregivers (ICs) has become critically important to community and long-term care systems.^1^ ICs play crucial roles in healthcare systems globally, providing first-line care and support to individuals in need and helping to contain long-term care costs.^2–4^ They are defined as individuals, often family members, friends, or neighbours, who provide unpaid care and support to people with chronic illnesses, disabilities, or other long-term health needs. These caregivers assist with activities of daily living (ADLs), clinical procedures, emotional support, and coordination of care, often stepping in where formal healthcare services are limited or unavailable.^5^ They contribute significantly to older people’s health outcomes, quality of life and well-being.^2–4^ They also commonly take on the role of coordinating and overseeing complex medical procedures, despite not having the formal training and expertise of healthcare professionals.^6^

In OECD countries, approximately 13.5% of individuals aged 50 and over provide informal care, with women representing the majority at 61.2%.^7^ As the population continues to age, the pool of people requiring care is projected to grow, potentially increasing the pressure on a relatively limited group of available caregivers.^8^,^9^ Growing reliance on informal caregiving can be attributed to a shortage of long-term care facilities and cultural norms emphasising intergenerational and familial support.^1^ Many countries are also undergoing a shift away from institutionalised care towards more home- and community-based services that are either formally or informally provided by family and friends.^10^,^11^ This trend is influenced by budgetary constraints but also by many individuals’ preference to age in a familiar physical and social environment for as long as possible.^11^,^12^

## The Complex Challenges of Informal Caregiving for Home-Dwelling Older People

Despite fulfilling important roles in enabling older people to remain at home and containing long-term care costs, ICs make personal sacrifices and often lack adequate support.^13^,^14^ A recent systematic review indicated potential associations between informal caregiving and the onset of various mental and physical health conditions, including a higher occurrence of depression, anxiety, pain, hypertension and diabetes, and reduced quality of life.^13^ Results from a longitudinal population-based study of older people in Germany showed statistically significant higher incidences of severe stress, adjustment disorders, depression, spinal diseases and pain conditions among ICs than among non-caregivers.^15^ In a qualitative synthesis of ICs’ experiences of caring for older people, Rezende et al highlighted the conflicting feelings of the care process, showing the complexity of the caregiving experience and how ICs put their lives on hold to provide care and sought coping strategies, including formal and informal support and spirituality.^16^ Furthermore, the economic burdens on ICs are frequently underestimated despite evidence suggesting they incur substantial financial expenses and invest significant amounts of time.^17^ ICs often spend a significant portion of their income, short-term savings, and even long-term savings to care for older family members at home.^18^ These expenses commonly include transportation, medical equipment, home care supplies, and both prescription and over-the-counter medications—costs that can quickly strain household budgets and undermine ICs’ sense of financial security and overall well-being.^17^,^19^ Caregiving also demands a considerable time commitment, often equivalent to a full-time job.^17^,^18^ As a result, many ICs experience job loss, reduced work hours, limited career advancement, or even complete withdrawal from the labor market.^19^ Longer hours of caregiving are closely associated with increased financial strain and reduced economic well-being.^19^

In low- and middle-income countries, financial pressures are even more significant, with caregivers frequently pausing work and covering costs for medications, food, and transport.^20^

These challenges must be understood within the broader cultural and structural contexts that shape caregiving practices. In many low- and middle-income countries, informal caregiving is shaped by both structural limitations in healthcare systems and cultural norms.^20^ Across regions such as Asia and Africa, caregiving for older people is often viewed as a moral duty and social obligation, rooted in traditions of filial responsibility and collective family care.^21^,^22^

In hospital settings, cultural expectations of family responsibility, combined with persistent shortages of healthcare personnel, result in the implicit transfer of essential care tasks to ICs.21 These may include feeding, bathing, administering medications, transporting patients, and providing emotional support and advocacy.21 While this practice mitigates understaffed health services, it simultaneously reinforces social norms around familial caregiving.^21^ As a result, ICs often function as vital—yet largely unacknowledged—actors within healthcare systems. Informal Caregiving’s Important Role in Safe Transitional Hospital-to-Home Care for Older People.

**Figure 1:**
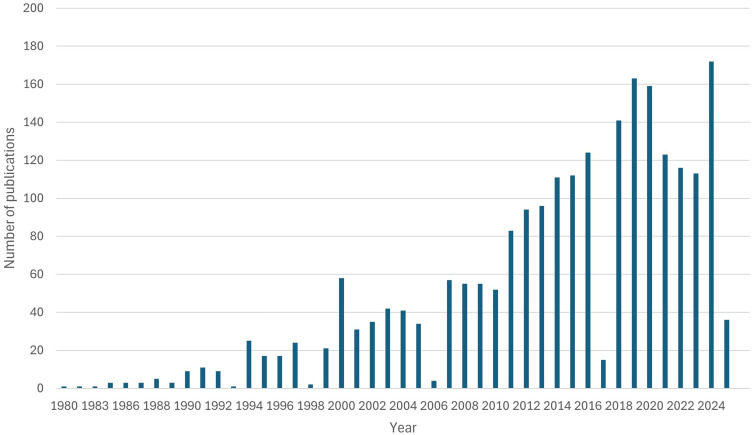
Papers referenced in Pubmed over the last 45 years on the role of ICs in transitional hospital-to-home care for older adults.

Research indicates that ICs play a crucial role in transitional hospital-to-home care for older people.^23^ Due to the growing recognition of the challenges faced by older people during transitions from hospital to home, together with the impact of these transitions on their well-being and the healthcare system, the role of ICs has emerged as a key focus in care practices. Their involvement in transitional care has also become the focus of considerable research interest and media attention over the past two decades. Figure 1 illustrates the increasing number of publications indexed in PubMed over the last 45 years on the role of ICs in transitional hospital-to-home care for older adults, based on a bibliometric analysis conducted by the authors (search strategies are provided in Tables S1 and S2, Supplementary File 1). However, the mechanisms behind effective transitional support remain insufficiently defined. The present paper provides a timely addition to the expanding body of evidence, offering new perspectives on the understanding of ICs’ roles—well beyond the provision of basic assistance—in ensuring safe and effective transitions for older people from hospital to home.

ICs offer tangible support, help and personal care, primarily in maintaining older people’s independence. They support older people by using their intimate understanding of the individuals’ priorities to make essential decisions in transitional care—the process of moving patients and their care from one environment to another.^23^ Ensuring safe, high-quality care transitions depends on the collaborative efforts of the healthcare system, hospitals, professionals, patients and their ICs.^23–25^ Safe transitional care contributes to integrated care, a broader approach aimed at providing seamless, comprehensive services to meet older people’s complex health needs by coordinating different services across the healthcare continuum.^26^ Findings from a case study analysis about integrating ICs in delirium prevention care for hospitalised older people indicated that they played crucial roles in providing both physical and psychological support.^27^ They ensured older people’s safe mobility by assisting them in their journeys, and they helped them in the activities of daily living. They were also involved in older people’s post-discharge plans, fostering their hopes of a swift return home.^27^

Aase et al’s meta-synthesis to assess quality and safety in care transitions emphasised the importance of patient-centred care, effective communication, collaboration, competency, accountability, and the cultural and spatial components of care environments.^25^ These components are crucial for facilitating interactions between patients, professionals and ICs and, consequently, for the provision of safe, timely, seamless care for patients moving between different settings and professional groups.^25^ Transitional care encompasses discharge planning—a tailored plan assessing the patient’s health and social needs prior to leaving hospital. Discharge planning should ensure a smooth transition from hospital to home (or another setting) by improving the coordination of post-discharge services.^28^ Safe transitional care, therefore, requires proactive measures that start well before a patient is discharged.^29^ Given that ICs play vital roles in providing necessary care, supporting recovery and independence, and facilitating early hospital discharge, they should be recognised as key partners throughout hospital stays and care transition periods.^30^,^31^ Yet, their presence and actions often go unnoticed within healthcare systems; the care they provide during a hospital stay is unacknowledged, and they are not recognised as system users.^23^ Consequently, hospitalisation can be stressful for ICs, increasing caregiver burden.^32^

Donzé et al’s study, explored the effectiveness of a transitional care intervention designed for higher-risk medical patients and aimed at reducing rates of 30-day unplanned readmissions and mortality.^33^ Their randomised clinical trial enrolled 1386 high-risk patients (mean age=72; SD=14 years) across four hospitals in Switzerland. The study observed no statistical difference in 30-day unplanned readmissions or mortality. The intervention also found no effects on healthcare use after discharge, patient satisfaction regarding their care transition, or costs associated with readmissions.^33^ The authors suggested that the absence of any significant effects might be due to the intervention’s limited scope, which did not include ICs or community support and focused solely on symptom monitoring and management. They concluded that ICs’ involvement might be particularly important due to patients failing to remember their discharge information.^33^

We conclude that ICs have a key role to play in ensuring safe transitional hospital-to-home care for older people, but their involvement may vary and must be considered by healthcare professionals and institutions, researchers and policymakers.

## Gaps in the Collaboration with Informal Caregivers Involved in Older People’s Transitional Care

In their recent systematic review of older people’s and ICs’ experiences, views and needs in transitional care decision-making, Kraun et al revealed ICs’ expectations regarding their involvement in discharge planning.^23^ ICs reported feeling insufficiently included in the care transition decision-making for those they cared for.^23^ Their involvement in discharge planning discussions or invitations to participate with older people in medical consultations were uncommon. They also experienced a loss of control, particularly regarding medication management, regimen restrictions, home care services and other pertinent information for care transitions.^23^ This was also highlighted in a mixed-methods study involving the ICs of home-dwelling polymedicated older people after hospital discharge.^34–36^ ICs perceived hospital discharge as desirable but distressing. They felt that they were insufficiently listened to and consulted in transitional care medication-related decisions. They described poor communication between hospital units and few opportunities for discussions with hospital professionals.^34^,^35^ Some ICs described the burdens of ensuring effective care transitions and home healthcare services. Older people, ICs and healthcare professionals have all called for better communication, collaboration and coordination between the professionals involved in transitional hospital-to-home care for older people.^35^,^36^

Hengelaar et al’s thematic synthesis on collaborations between healthcare professionals and ICs—from the professionals’ perspectives—described complex interactions. It highlighted that most problems stemmed from communication challenges, likely due to differing backgrounds and expectations about caregiving. Their work suggested that healthcare professionals needed to adapt their approaches when working with ICs, and they considered dialogue with ICs crucial to bridging the gaps in expectations and collaborative experiences. To achieve this, healthcare professionals suggested integrating information on collaborating with ICs into their education curriculum.^37^

Gaps hindering communication and collaboration in ICs’ involvement in transitional care could be responsible for older people’s adverse health outcomes. Insufficient participation in transitional care could discourage ICs from actively partaking in decision-making processes or from adopting roles as older people’s supporters, advocates or representatives.^30^,^38^ This could contribute to discharge planning ineffectiveness, premature hospital readmissions and undesirable admissions into long-term residential care facilities.^1^,^39^

## Strategies to Support Informal Caregivers for Safer Transitional Care for Older People

Although several studies have explored ICs’ experiences during transitional care, relatively little is known about how to effectively involve them in safer transitional hospital-to-home care for older people. However, the literature proposes several concrete directions for reform and practical strategies to address these issues.

One key strategy involves raising awareness among professional caregivers about the crucial role played by ICs in transitional care.^40^ Incorporating informal caregiving into healthcare education could help bridge gaps in expectations and foster more effective collaboration between professional and ICs during care transitions.^41^ Despite this, the majority of existing studies focus on training ICs, while limited attention is given to preparing healthcare professionals to engage and collaborate effectively with them. Further research is needed to develop clear guidelines and evidence-based training approaches.

Another key area of focus for safer transitional care is improving communication and information transfer. Effective information exchange and active engagement of patients and ICs have been identified as core elements for successful care transitions. Several studies emphasize the need for better communication, collaboration, and coordination between healthcare professionals and ICs.^27^,^34^ Suggested practical improvements include refining communication processes and ensuring that ICs’ information is accurately reflected in electronic health records. Several national and international initiatives have been launched in this regard. For instance, the Caregiver Advise, Record, Enable (CARE) Act, adopted by 42 states of the USA in 2022, mandates hospitals to record ICs’ details in patients’ electronic health records, notify ICs about patients’ hospital discharges and provide ICs with the knowledge and skills needed for safe, efficient transitions.^42^ North Carolina even introduced a hotline specially for older inpatients’ ICs, facilitating immediate access to family consultants for inquiries.^43^ A third strategy involves formalizing IC involvement and recognition. There is growing consensus on the need to foster structured partnerships with ICs and integrate them into decision-making processes throughout the hospital stay and care transitions.^23^,^29^,^31^ Recognizing ICs’ skills and acknowledging them as key partners is vital, especially since their contributions often go unnoticed.^23^ Notably, the lack of IC involvement has been cited as a reason for the absence of significant effects in some transitional care interventions, indicating that their participation is not only desirable but necessary for effectiveness.^29^,^33^ Wieczorek et al highlighted disparities in the support available to ICs across the EU, with countries like Sweden, Denmark, and the Netherlands offering more holistic and integrated systems.^12^

Finally, leveraging technology and support systems is a promising approach to strengthen support for ICs. Evidence shows that ICs frequently use digital tools—particularly smartphones—to seek health-related information, coordinate household and care tasks, communicate with family members and healthcare professionals, and connect with peers through online forums.^44^ These forums are especially valued for maintaining social ties, which are often weakened by the constraints of caregiving. The findings highlight that providing clear, reliable, and accessible digital content is a strategic lever for strengthening ICs’ knowledge, confidence, and self-management abilities.^44^ Countries like Sweden exemplify this by providing innovative digital aids—such as e-care, e-health services, peer support networks, and online learning resources—that help ICs manage their responsibilities more effectively, especially during care transitions.^12^ This has generated growing interest in the development of digital platforms that offer training, well-being resources, and communication tools tailored to ICs’ needs. However, despite this growing interest, the available evidence reveals significant gaps regarding the effectiveness of mobile apps designed to assist ICs.^45^ There is also a lack of evidence-based guidelines for developing and evaluating such information technology applications.^46^ Indeed, few studies have developed these technologies in the specific sensitive context of transitional hospital-to-home care for older people.^45^ Moreover, these welfare technologies do not entirely meet the needs of a significant proportion of ICs worldwide who are unfamiliar with such tools.^47^,^48^ Furthermore, actively involving ICs in the development of these technologies is crucial to ensure that digital solutions effectively address their real-world needs and challenges.^44^

The principles of recognizing, educating, and collaborating with ICs are universally valuable, but their implementation necessitates an understanding of specific cultural, social, and economic dynamics.

## Theoretical Approaches

To effectively support ICs in ensuring safer transitional care for older people, two complementary theoretical approaches should be considered: safe transitional care and active engagement of ICs in the care process. Achieving optimal transitions in care requires attention to *seven essential intervention categories*: medication management, transition planning, patient and family engagement/education, information transfer, follow-up care, healthcare provider engagement, and shared accountability across professionals and organisations.^49^ Models and interventions like Better Outcomes for Older people through Safe Transitions (BOOST), the Care Transitions Intervention (CTI) and the Transitional Care Model (TCM) were specifically designed to enhance continuity during transitional care. They have shown significant benefits for high-risk groups, notably older people, who not only face more frequent hospitalisations and transitions between care settings but also present higher risks of complications, readmissions, and increased post-discharge morbidity and mortality.^24^,^50^ A systematic review on the TCM for older people with multimorbidity discharged from hospital to community dwelling, indicated that early home visits (eg, within 24–48 hours post-discharge) and continuous communication, were associated with reduced readmission rates.^51^ Key components identified as having an important role in reducing readmission rates include assessing and managing symptoms, educating and promoting self-management, maintaining relationships, fostering coordination and engaging patients and ICs.^51^ The review also underscored the importance of addressing both hospital and community settings in transitional care interventions, as understanding the “real” home environment reveals unique barriers and supportive factors not always apparent from a hospital perspective.^51^ Schulz et al’s framework categorises caregiving interventions various across three levels: individual, organisational and societal.^52^ At the individual level, interventions target both ICs and care recipients, addressing information needs, coping strategies, communication skills, and access to support through approaches such as psychoeducation, self-management, mindfulness, physical activity, and cognitive behavioral therapy.^52–54^

At the organizational level, healthcare systems and workplaces significantly shape the caregiving experience.^52^ Nurse-led care coordination, caregiver assessments, and tailored support plans enhance care delivery.^55^,^56^ Employment conditions, including caregiver-friendly policies (eg, flexible hours, remote work, job protection), can mitigate caregiver burden and promote sustainable caregiving.^57^

At the societal level, structural and policy factors are central. Accurate data on caregiving prevalence informs policy, while stronger national support systems correlate with better outcomes for ICs.^12^ Key actions include formal integration of ICs into health and social care systems and public education to prepare citizens for caregiving.^12^,^52^ Interventions should be evaluated based on broad societal outcomes, such as reduced healthcare use and delayed institutionalization.^52^

## Research Gaps and Future Research

There is a burgeoning body of evidence recognising and addressing the challenges ICs face and highlighting their indispensable role in the healthcare continuum. However, despite these advances, there is a lack of national and international evidence about how to effectively involve ICs in the specific context of transitional hospital-to-home care for older people. As many countries increasingly recognize the role of ICs in health and social care systems, there is a need for specific guidelines and training for professional caregivers on how to collaborate with ICs effectively.^12^,^42^,^43^,^58^,^59^ Another critical challenge is the scarcity of evidence describing the extent to which ICs’ involvement in transitional care impacts older people’s outcomes, such as functional status, emergency department visits and readmissions after hospital discharge.^29^

Further research is required to empower ICs in transitional care decision-making and to develop means for collaborative partnerships between ICs and healthcare professionals. Such knowledge should contribute to tangible interdisciplinary clinical improvements, such as increasing professional caregivers’ awareness of ICs’ roles, ensuring ICs’ information is accurately reflected in electronic health records, refining communication processes, and meeting the specific needs for safer transitional hospital-to-home care for older people.

## Conclusion

Informal caregivers (ICs) are indispensable partners in providing transitional hospital-to-home care for older people, playing an essential role in supporting safety and continuity of care. Despite their crucial contributions, significant gaps persist in their formal recognition and integration into discharge planning and decision-making processes, leading to feelings of insufficient inclusion, poor communication, and limited opportunities for discussion with hospital professionals. These persistent gaps in collaboration are detrimental, potentially compromising patient safety, continuity of care, and contributing to adverse health outcomes, including premature hospital readmissions. Addressing these systemic challenges requires proactive measures, including integrating ICs into professional training, refining hospital discharge protocols, and fostering collaborative partnerships. By doing so, healthcare systems can enhance the quality and safety of transitional care, alleviate caregiver burden, and promote better long-term health trajectories for older people. Further investigation is required to address these critical gaps in evidence and to develop means for collaborative partnerships, empowering ICs, and ultimately contributing to tangible interdisciplinary clinical improvements for safer transitional hospital-to-home care for older people.

## Abbreviation

IC, informal caregiver.
